# The impact of non-structured PSA testing on prostate cancer-specific mortality on New Zealand Māori men

**DOI:** 10.1007/s00345-024-05260-2

**Published:** 2024-10-03

**Authors:** Bashar Matti, Cindy H. Nguyen, Mataroria P. Lyndon, Kamran Zargar-Shoshtari

**Affiliations:** 1 Department of Surgery, Faculty of Medical and Health Sciences, University of Auckland, Auckland, New Zealand; 2 Faculty of Medical and Health Sciences, Centre for Medical and Health Science Education, University of Auckland, Auckland, New Zealand; 3 Department of Surgery, Counties Manukau District Health Board, Auckland, New Zealand

**Keywords:** Prostate-specific antigen, Early detection of cancer, Mass screening, Prostatic neoplasm, Health inequities

## Abstract

**Objectives:**

To assess the impact of differences in Prostate-Specific Antigen (PSA) testing rates on prostate cancer (PCa) diagnosis and PCa-specific mortality among Māori men in a New Zealand (NZ) population.

**Patients and Methods:**

Māori men aged 40 years or older, without a history of PCa, with a PSA test between 2006 and 2018 were included. The cohort was divided into two groups; the “screened group” (ScG) consisting of men who had at least one PSA test every four years or less, and the “non-screened group” (non-SG). We measured the rate of cancer diagnoses and used competing risk analysis to assess survival.

**Results:**

The study cohort included 63,939 Māori men, with 37,048 (58%) in the ScG. PCa was more frequently diagnosed in the ScG (3.7% vs. 3.0%, *P* < 0.001). A higher proportion of high-grade cancers were found in the non-SG (32.7% vs. 25.6%, *P* = 0.001). The 10-year cancer-specific survival was significantly higher in the ScG (99.4% vs. 98.5%, *P* < 0.001). In a multivariable risk model, PSA testing frequency was an independent predictor of PCa mortality. (HR 2.43, [95% CI 1.97–3.01], *P* < 0.001).

**Conclusions:**

In a cohort of only Māori men, lower PSA testing rates were associated with a higher risk of PCa-related death. Therefore, regular PSA testing for Māori could improve cancer-specific survival among Māori men. Regular PSA testing should be considered a priority area for improving PCa survival in this population.

**Supplementary Information:**

The online version contains supplementary material available at 10.1007/s00345-024-05260-2.

## Introduction

New Zealand (NZ) has one of the highest incidences of prostate cancer (PCa) in the world (105.2 per 100 000) [1]. Currently, there is no population-based screening program in NZ. Instead there is widespread opportunistic Prostate-Specific Antigen (PSA) testing in NZ and subsequently high rates of PCa detection. Data shows that not all men in NZ have equitable access to PSA testing. In particular, Māori men are significantly less likely to receive a PSA test and are less likely to have regular PSA testing [2]. This lower rate of PSA testing may explain the lower incidence of PCa seen in NZ Māori compared to the NZ European population [1].

The European Randomized Study of Screening for Prostate Cancer (ERSPC) trial has shown that repeated cycles of PSA testing reduces PCa mortality [3]. On this basis, we hypothesised that the lower rates of PSA testing in Māori are a direct contributor to the worse PCa-specific survival seen in this population. However, to minimise social and cultural confounders that may affect PCa survival, we aimed to assess the impact of regular PSA testing on PCa outcomes (diagnosis and mortality) in a cohort of only Māori men [4].

## Methods

### Study population

The study population constituted all Māori men who had a PSA test between 1 January 2006 and 1 January 2018. Men with a prior diagnosis of PCa were excluded and assumed to have PSA tests for disease monitoring. We also excluded men younger than 40 because PSA is generally not utilised in PCa screening among these men. Only one PSA test per calendar year was considered in the dataset for each patient to avoid potential bias related to repeated testing in a short period, usually triggered by an abnormal result.

### Data sources

Data were obtained from three prospectively maintained national databases: (1) the NZ Ministry of Health laboratory warehouse containing all laboratory test results; (2) the New Zealand Cancer Registry (NZCR) which records all new cancer diagnoses; and (3) the National Mortality Collection which provides data on causes of death derived from the weekly collection of death certificates, coroner reports, and hospital records. Data linkage was feasible using the National Health Index (NHI), a unique alphanumeric identifier assigned by the Ministry of Health to each health user in NZ. Ethnicity was self-reported and captured at the point of enrolment to a general practice or at after presentation to hospital. This was automatically linked to NHIs collected. All PSA test results, test dates, and cancer diagnoses for each NHI were collated in our study database. Low-, intermediate- and high-grade cancer corresponded to Gleason 6, Gleason 7 and Gleason 8 or higher, respectively. Socioeconomic status was measured based on the New Zealand Index of Deprivation (NZDep index) using nine variables derived from the national Census [5].

### Outcomes measured

The cohort was divided into two groups. First, Māori men with high levels of PSA testing were classified as the “screened group” (ScG). The ScG was defined as men who had two or more PSA tests, with a rate of one test every four years or less. The second cohort was the “non-screened group” (non-SG) which included all other men. The outcomes of interest were: (1) PCa diagnosis, including cancer grade and; (2) PCa-specific mortality.

### Data management and statistical analysis

Data analysis and management were conducted using SPSS 25.0. Descriptive statistics were reported as medians with an interquartile range (IQR) or crude numbers with percentages. The study groups were compared using Mann-Whitney U non-parametric or chi-squared tests.

The risk of PCa diagnosis during the study period was estimated with univariable and multivariable binary logistic regression models and reported as an odds ratio (OR) with a 95% confidence interval (CI). For cancer-specific survival (CSS), we used the cumulative incidence for competing death events to account for causes of death other than PCa. Similarly, the risk of death from PCa was estimated with univariable and multivariable Fine-Gray proportional hazard regression and reported as a hazard ratio (HR) with a 95% CI. Time in these calculations was measured in days from the date of enrolment in the study (first PSA test in the study period) to the date of death or study termination. For all calculations, a *P*-value < 0.05 was considered statistically significant.

## Results

Between 1 January 2006 and 1 January 2018, there were 2,941,916 PSA tests performed on 825,259 men in NZ. Of these, 176,820 PSA tests were performed in 63,939 (7.9%) men who identified as Māori and were included in the final analysis.

Of these, 37,084 (58%) Māori men were included in the ScG. These men had a total of 147,481 PSA tests during the study period. The non-SG comprised 26,855 Māori men who received 29,339 PSA tests.

Men in the ScG were slightly older than men in the non-SG (54 vs. 51 years, *P* < 0.001). ScG men received a median of three tests per person (IQR 3). Table 1 summarises relevant baseline characteristics in the two groups.

**Table 1:** Cohort baseline characteristics (*n =* 63,939)

Characteristic	Total cohort	Non-screened group	Screened group	*P* value
No. men, *n* (%)	63,939	26,855 (42)	37,084 (58)	N/A
No. PSA tests, *n* (%)	176,820	29,339 (16.6)	147,481 (83.4)	N/A
Median age (yr), *n* (IQR)	53 (13)	51 (14)	54 (13)	< 0.001
Median NZ Dep (decile)	8 (5)	8 (4)	8 (4)	< 0.001
Median No. PSA tests per person, *n* (IQR)	2 (3)	1 (0)	3 (3)	< 0.001
Median cost of PSA testing (NZD), *n* (IQR)	22 (28.5)	11 (0.1)	33.22 (32.8)	< 0.001
Follow-up (yr), *n* (IQR)	6.27 (5.93)	3.57 (5.41)	7.78 (4.64)	< 0.001
Prostate Cancer, *n* (%)	2,169 (3.4)	794 (3.0)	1,375 (3.7)	< 0.001

This table compares the baseline characteristics between non-screened group and screened group over a 12 year period. P values were The study groups were compared using Mann-Whitney U non-parametric testing or chi-squared test

IQR = interquartile range; NZ Dep = New Zealand Deprivation Index; NZD = New Zealand Dollar; PSA = Prostate-Specific Antigen

### Cancer diagnosis

A total of 2169 men were diagnosed with PCa in the cohort, with a significantly higher proportion of cancers diagnosed in the ScG (3.7% vs. 3.0%, *P* < 0.001). Following adjustments for age and NZDep index, the risk of cancer detection was 15% higher in the ScG (OR 1.15, [95% CI 1.05–1.26], *P* = 0.002) (Table 2).

**Table 2:** Univariable and multivariable logistic regression risk model predicting prostate cancer diagnosis in the cohort

Variable	Univariable			Multivariable		
	OR	95% CI	*P*value	Adjusted OR	95% CI	*P*value
Age	1.08	1.07–1.08	< 0.001	1.08	1.07–1.08	< 0.001
NZDep index	1.0	0.98 – 1.02	0.986	0.97	0.96–0.99	< 0.001
Non-screened group	Ref	Ref	-	Ref	Ref	-
Screened group	1.26	1.16–1.38	< 0.001	1.15	1.05–1.26	0.002

This table demonstrates factors associated with prostate cancer diagnosis using univariable and multivariable logistic regression. Those in the screened group were more likely to be diagnosed with prostate cancer (HR 1.15)

OR = odds ratio; CI = confidence interval; NZDep index = New Zealand area-based deprivation index

Within the entire cohort, 750 (38%) of diagnosed cancers were low-grade, 666 (33.8%) were intermediate, and 556 (28.2%) were high-grade. Men in the non-SG were significantly more likely to be diagnosed with high-grade PCa (32.7% vs. 25.6%, *P* = 0.001). The diagnosis rate of low-grade cancer was higher in the ScG (40.5% vs. 33.8%, *P* = 0.003). There was no statistically significant difference in the proportion of intermediate risk PCa diagnosed between the two groups (33.4% vs. 34.0%, *P* = 0.804) (Supplementary Table 1).

### Survival analysis

**Fig. 1:**
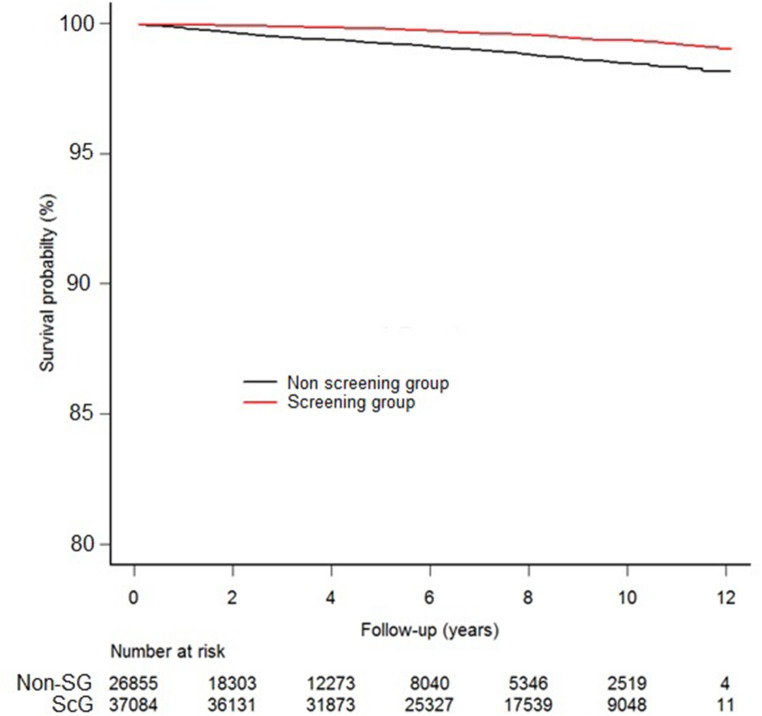
Cancer-specific survival (CSS) among the non-screening group and screening group over 10 years. The screening group had higher CSS than the non-screening group (99.4% vs. 98.5%, *P* < 0.001) at 10 years. The CSS was calculated using the cumulative incidence for competing death events. The Gray test was used to compare groups

During the study period, 359 men died of PCa, with 196 (54.6%) deaths occurring in the non-SG. The median follow-up duration was 6.27 (IQR 5.93) years for the entire cohort, with 3.57 (IQR 5.41) years in the non-SG and 7.78 (IQR 4.64) years in the ScG (*P* < 0.001). Median PCa-specific survival was not reached in either group. However, the estimated 10-year CSS was significantly higher in the ScG (99.4% vs. 98.5%, *P* < 0.001) (Fig. 1).

In a multivariable competing risk analysis model assessing PCa-specific survival, being in the ScG was an independent predictor of survival. Māori men in the non-SG were more than twice as likely to die from PCa compared to Māori men in the ScG (HR 2.43, [95%CI 1.97–3.01], *P* < 0.001). Age was also an independent predictor for PCa survival (HR 1.11, [95% CI 1.11–1.13], *P* < 0.001). However, the NZDep index did not significantly contribute to PCa-specific survival in the model (*P* = 0.822) (Table 3).

**Table 3:** Univariable and multivariable fine-Gray competing risk model predicting prostate cancer death in the cohort

Variable	Univariable			Multivariable		
	HR	95% CI	*P* values	Adjusted HR	95% CI	*P* value
Age	1.12	1.11–1.13	< 0.001	1.12	1.11–1.13	< 0.001
NZDep index	1.06	1.02–1.11	0.004	1.00	0.96–1.05	0.822
Screened group	Ref	Ref	-	Ref	Ref	-
Non-screened group	2.75	2.25–3.36	< 0.001	2.43	1.97–3.01	< 0.001

This table shows factors predicting prostate cancer-related death using univariable and multivariable analysis. The non-screened group had an increased risk (HR 2.43) of prostate cancer-related death when compared to the screened group (reference group)

CI = confidence interval; HR = hazard ratio

## Discussion

This study has demonstrated that Māori men who undergo regular PSA testing were less likely to die from PCa compared to Māori men who did not undergo regular testing. Additionally, men who had regular PSA testing were more likely to be diagnosed with PCa but less likely to have a high-grade disease at diagnosis. This may explain the better PCa survival for these men. These findings are consistent with international data on opportunistic and regular PSA screening [3, 6]. The findings of this study are unique, and to our knowledge, this is the only study to assess the “real-world” impact of PSA testing on PCa survival specifically in a Māori population.

In a previous study, we reported that on average, Māori men received 28% fewer PSA tests than non-Māori men over ten years. The same study also showed that Māori men were far less likely to be offered regular PSA tests across all age groups as part of an opportunistic PCa risk assessment [7]. Furthermore, our group has previously concluded that PCa-specific survival in NZ is significantly lower for Māori men compared to non-Māori [7, 8]. We reported that the 10-year CSS for Māori was 71.5% compared to 79.3% for Europeans, 83.9% for Asians and 76.3% for Pacific Island men. This corresponded to a 44% higher risk of PCa death for Māori men compared to Europeans [8].

The current study has shown a strong association between the rate of PSA testing and PCa-specific survival with men in the non-SG at least twice as likely to die from PCa compared to the ScG. One of the strengths of this study is that only Māori men were included. This has minimised potential confounding factors which might impact PSA testing, diagnosis and survival. These factors include cultural barriers to PSA testing, and treatment-related factors [4, 7].

PSA screening has been shown to improve PCa-specific survival in large randomised studies, and the relationship between PSA testing and PCa survival is not a new finding [3]. The ERSPC clearly showed a survival advantage at 16 years of follow-up. Additionally, analysis of the same study has demonstrated that within the study population, socioeconomic factors seem to impact the rate of PSA testing, subsequent PCa diagnosis and PCa survival [9, 10]. Our study confirms that these findings apply to the NZ population.

Men in the non-SG were younger (54 vs. 51 years). Therefore, they may not have been eligible for regular testing since most practitioners start to offer testing to men in their 50s. However, it should be noted that the ScG had a median of three cycles of testing, which suggests that the three-year median difference in the age of the groups does not explain the significant variation in testing. Also, a larger proportion of the non-SG died of PCa, indicating that testing in Māori men effectively reduces PCa mortality. Therefore, PSA testing should perhaps start at an earlier age in Māori.

We found the increasing NZDep index was associated with a slight reduction in PCa diagnosis. This aligns with other prior studies which suggests socioeconomic status has an impact on PSA testing regardless of ethnicity [4, 7]. 

Another important consideration in our study was the definition of regular PSA testing. It is accepted that only recurrent PSA testing at regular intervals results in improved PCa outcomes and that low-intensity and infrequent testing has no positive impact on the outcomes [11, 12]. Men in the ScG had a median of three tests during the 12-year study period, which fell well within the accepted definitions for PSA screening. Pakarainen et al. reported that in the Finnish arm of the ERSPC trial, men who attended two or three rounds of screening experienced improved PCa survival [11].

The CSS was significantly lower in the non-SG. This indicates that in real-world settings, regular PSA testing significantly impacts PCa survival in Māori men. Our study suggests that the previously reported disparities in PCa outcomes in Māori compared to non-Māori may be addressed by ensuring equitable access to regular PSA testing for Māori [13]. As seen in Fig. 1, most PCa deaths in the non-ScG occurred within six years following diagnosis, inferring that this was primarily death from aggressive high-risk disease. The discrepancy in median follow-up time between the non-SG and ScG was an interesting finding. Men in the non-SG may have fewer PSA tests because of reduced engagement with health care services or competing comorbidities leading earlier to loss to follow-up. However, we cannot comment with certainty the reason for this due to the retrospective nature of our study.

Our study has also demonstrated that regular PSA testing was associated with an increased risk of PCa diagnosis. This aligns with international studies and represents the well-established potential harm of overdiagnosis in PCa screening [14, 15]. However, the difference in the rates of low-risk disease between the ScG and non-SG groups was merely 7.7%. With advancements in cancer detection and the broader applicability of active surveillance, the balance in this cohort may well be leaning toward the survival benefits achieved through regular PSA testing [16].

Our study has some limitations. Firstly, the study is a retrospective analysis of prospectively collected data. We cannot ascertain if the two groups were identified as no data on comorbidities was available in the dataset. The baseline PSA level between the two groups was not compared. However, all men in this cohort had a PSA test and therefore were deemed “fit enough” for PCa screening by their primary healthcare providers. Secondly, there were no data on PCa disease stage at diagnosis, the treatment received or the length of follow-up between the study groups, which could impact patient outcomes. This information was generally poorly documented in the NZCR for PCa. In addition, our study did not identify causes for the different rates of PSA testing among Māori men. However, despite these limitations, we found significant differences in the outcomes.

## Conclusion

In this study of only Māori men, lower PSA testing rates were linked to a higher risk of PCa-related death. Therefore, regular PSA testing for Māori could be an important step towards improving cancer-specific survival among Māori men. Inequitable access to PSA testing between Māori and non-Māori men has previously been demonstrated. Ensuring equitable PSA testing rates for Māori should be considered a step towards improving cancer outcomes for Māori men.

## Electronic supplementary material

Below is the link to the electronic supplementary material.

Supplementary Material 1

## Data Availability

The data supporting these findings were provided by the New Zealand Ministry of Health following ethical approval. De-identified data can be obtained from the primary author, BM, upon reasonable request and with appropriate ethical clearance.

## References

[1] Matti B, Chapman D, Zargar-Shoshtari K (2021) Ethnic and regional differences in the temporal trends of prostate cancer incidence and mortality in New Zealand. ANZ J Surg 91(12):2806–2816 34676954 10.1111/ans.17263

[2] Matti B, Zargar-Shoshtari K (2020) Opportunistic prostate cancer screening: a population-based analysis. Urol Oncol 38(5):393–400 31952998 10.1016/j.urolonc.2019.12.009

[3] Hugosson J, Roobol MJ, Mansson M, Tammela TLJ, Zappa M, Nelen V et al (2019) A 16-yr follow-up of the European randomized study of screening for prostate Cancer. Eur Urol 76(1):43–51 30824296 10.1016/j.eururo.2019.02.009 PMC7513694

[4] Moses KA, Zhao Z, Bi Y, Acquaye J, Holmes A, Blot WJ et al (2017) The impact of sociodemographic factors and PSA screening among low-income Black and White men: data from the Southern Community Cohort Study. Prostate Cancer Prostatic Dis 20(4):424–429 28695916 10.1038/pcan.2017.32 PMC5861729

[5] Atkinson J, Salmond C, Crampton P (2014) NZDep2013 index of deprivation. Department of Public Health, University of Otago, Wellington

[6] Schröder FH, Hugosson J, Roobol MJ, Tammela TL, Zappa M, Nelen V et al (2014) Screening and prostate cancer mortality: results of the European Randomised study of screening for prostate Cancer (ERSPC) at 13 years of follow-up. Lancet 384(9959):2027–2035 25108889 10.1016/S0140-6736(14)60525-0 PMC4427906

[7] Matti B, Lyndon M, Zargar-Shoshtari K (2021) Ethnic and socio-economic disparities in prostate cancer screening: lessons from New Zealand. BJU Int 128(Suppl 3):11–17 32599662 10.1111/bju.15155

[8] Matti B, Zargar-Shoshtari K (2021) Prostate cancer outcomes disparities: Population survival analysis in an ethnically diverse nation. Urol Oncol 39(6):367 e19- e26 10.1016/j.urolonc.2021.02.023 33858746

[9] Kilpelainen TP, Talala K, Raitanen J, Taari K, Kujala P, Tammela TLJ et al (2016) Prostate Cancer and socioeconomic status in the Finnish randomized study of screening for prostate Cancer. Am J Epidemiol 184(10):720–731 27777219 10.1093/aje/kww084

[10] Tomic K, Ventimiglia E, Robinson D, Haggstrom C, Lambe M, Stattin P (2018) Socioeconomic status and diagnosis, treatment, and mortality in men with prostate cancer. Nationwide population-based study. Int J Cancer 142(12):2478–2484 29363113 10.1002/ijc.31272 PMC5947133

[11] Pakarainen T, Nevalainen J, Talala K, Taari K, Raitanen J, Kujala P et al (2019) The number of screening cycles needed to reduce prostate Cancer mortality in the Finnish section of the European Randomized study of prostate Cancer (ERSPC). Clin Cancer Res 25(2):839–843 30322875 10.1158/1078-0432.CCR-18-1807

[12] Martin RM, Donovan JL, Turner EL, Metcalfe C, Young GJ, Walsh EI et al (2018) Effect of a low-intensity PSA-Based screening intervention on prostate Cancer mortality: the CAP Randomized Clinical Trial. JAMA 319(9):883–895 29509864 10.1001/jama.2018.0154 PMC5885905

[13] Matti B, Lyndon M, Zargar-Shoshtari K (2021) Ethnic and socio‐economic disparities in prostate cancer screening: lessons from New Zealand. BJU Int 128:11–17 32599662 10.1111/bju.15155

[14] Fenton JJ, Weyrich MS, Durbin S, Liu Y, Bang H, Melnikow J (2018) Prostate-specific antigen–based screening for prostate cancer: evidence report and systematic review for the US Preventive Services Task Force. JAMA 319(18):1914–1931 29801018 10.1001/jama.2018.3712

[15] Schröder FH, Hugosson J, Roobol MJ, Tammela TLJ, Ciatto S, Nelen V et al (2009) Screening and prostate-Cancer mortality in a randomized European study. N Engl J Med 360(13):1320–1328 19297566 10.1056/NEJMoa0810084

[16] Stabile A, Giganti F, Rosenkrantz AB, Taneja SS, Villeirs G, Gill IS et al (2020) Multiparametric MRI for prostate cancer diagnosis: current status and future directions. Nat Reviews Urol 17(1):41–61 10.1038/s41585-019-0212-4 31316185

