# Supplementary material for: The impact of non-structured PSA testing on prostate cancer-specific mortality on New Zealand Māori men
Source: World J Urol. 2024 Oct 3;42(1):558. doi: 10.1007/s00345-024-05260-2 (PMC11449978; doi:10.1007/s00345-024-05260-2)
Supplement: Supplementary file 1 — Supplementary Material 1 [file 345_2024_5260_MOESM1_ESM.docx]

Supplementary Table 1. Cancer grade group classification in the screened group and non-screened group

| Grade | Cohort, *n* (%) | Non-screened group, *n* (%) | Screened group, *n* (%) | *P* value |
| --- | --- | --- | --- | --- |
| Low | 750 (38.0 | 245 (33.8) | 505 (40.5) | 0.003 |
| Intermediate | 666 (33.8) | 242 (33.4) | 424 (34.0) | 0.804 |
| High | 556 (28.2) | 237 (32.7) | 319 | 0.001 |
| Total* | 1972 | 724 | 1248 |  |
| Cancer grade groups at diagnosis for the non-screened and screened groups. Low-, intermediate- and high-grade cancer corresponded to Gleason 6, Gleason 7 and Gleason ≥8, respectively.  *Unknown grade = 197 cases (9.1%) | | | | |
